# Comparison of Quality of Recovery between Modified Thoracoabdominal Nerves Block through Perichondrial Approach versus Oblique Subcostal Transversus Abdominis Plane Block in Patients Undergoing Total Laparoscopic Hysterectomy: A Pilot Randomized Controlled Trial

**DOI:** 10.3390/jcm13030712

**Published:** 2024-01-25

**Authors:** Takanori Suzuka, Nobuhiro Tanaka, Yuma Kadoya, Mitsuru Ida, Masato Iwata, Naoki Ozu, Masahiko Kawaguchi

**Affiliations:** 1 Department of Anesthesiology, Nara Medical University, 840 Shijo-cho, Kashihara 634-8522, Nara, Japan; szk.tknr.0825@naramed-u.ac.jp (T.S.); nwnh0131@naramed-u.ac.jp (M.I.); drjkawa@naramed-u.ac.jp (M.K.); 2 Department of Anesthesiology, Ikeda City Hospital, 3-1-18 Jonan, Ikeda 635-8501, Osaka, Japan; kadoyayuuma@naramed-u.ac.jp; 3 Department of Anesthesiology, Yamatotakada Municipal Hospital, 1-1, Isonokita-cho, Yamatotakada 635-8501, Nara, Japan; miwata@naramed-u.ac.jp; 4 Institute for Clinical and Translational Science, Nara Medical University Hospital, 840 Shijocho, Kashihara 634-8522, Nara, Japan; nao.oz@naramed-u.ac.jp

**Keywords:** nerve block, pain management, pain, postoperative period, postoperative complications, regional anesthesia

## Abstract

Modified thoracoabdominal nerves block through a perichondrial approach (M-TAPA) provides a wide analgesic range. Herein, we examined the quality of recovery (QoR) of M-TAPA for total laparoscopic hysterectomy (TLH) compared with oblique subcostal transversus abdominis plane block (OSTAPB) and measured plasma levobupivacaine concentrations (PC_levo_). Forty female patients undergoing TLH were randomized to each group. Nerve blocks were performed bilaterally with 25 mL of 0.25% levobupivacaine administered per side. The primary outcome was changes in QoR-15 scores on postoperative days (POD) 1 and 2 from the preoperative baseline. The main secondary outcomes were PC_levo_ at 15, 30, 45, 60, and 120 min after performing nerve block. Group differences (M-TAPA—OSTAPB) in mean changes from baseline in QoR-15 scores on POD 1 and 2 were −11.3 (95% confidence interval (CI), −24.9 to 2.4, *p* = 0.104; standard deviation (SD), 22.8) and −7.0 (95% CI, −20.5 to 6.6, *p* = 0.307; SD, 18.7), respectively. Changes in PC_levo_ were similar in both groups. The post hoc analysis using Bayesian statistics revealed that posterior probabilities of M-TAPA being clinically more effective than OSTAPB were up to 22.4 and 24.4% for POD 1 and 2, respectively. In conclusion, M-TAPA may not be superior to OSTAPB for TLH.

## 1. Introduction

Total laparoscopic hysterectomy (TLH) is a common minimally invasive gynecological procedure; however, the postoperative, pneumoperitoneum, and spiritual distress, as well as somatic and visceral pain, can be intense [1,2]. Acute pain management in the early postoperative period is important to improve various outcomes, leading to the reduction of the incidence of chronic pain [3].

Peripheral nerve block, in particular transverse abdominis plane (TAP) block, is commonly used to manage postoperative pain in abdominal surgery. A recent systematic review does not recommend TAP block for laparoscopic hysterectomy due to the lack of consistent evidence [4,5]. This discrepancy may be attributed to the confusion of several types of TAP blocks, such as lateral TAP block (mid-axillary TAP block) [6], subcostal TAP block, 4-point TAP block, and oblique subcostal TAP block (OSTAPB). OSTAPB provides a wide range of analgesia from T7 to T12, is effective for 6–18 h postoperatively [7], and is effective in lower abdominal surgery, including TLH; however, OSTAPB requires multiple injections to achieve complete analgesia [7,8].

Modified thoracoabdominal nerves block through a perichondrial approach (M-TAPA) has received increasing attention due to its wide range of analgesic coverage of the trunk (i.e., T7–T12) with a single puncture per side and has been initially reported to provide a long-acting effect [9,10,11]. The efficacy of M-TAPA has been investigated in laparoscopic cholecystectomy and laparoscopic inguinal hernia repair in randomized controlled studies [12,13,14]; however, its efficacy in TLH has not been clarified.

Investigating the plasma concentration changes after nerve blocks is crucial for furthering the understanding of the prevention of systemic toxicity when using nerve blocks; however, no study has evaluated the plasma concentrations of local anesthetics after M-TAPA without additive agents [15]. M-TAPA has been confirmed to have a residual effect via a pinprick test after 24 h postoperatively, suggesting the possibility of obtaining overnight analgesia in some initial reports [9,11]. Therefore, we hypothesized that the pattern of changes in the plasma concentrations of local anesthetic in M-TAPA might differ from that of other fascial plane blocks. 

We hypothesized that M-TAPA would be more effective than OSTAPB for postoperative recovery, considering its range of efficacy and long-term durability. We designed a pilot randomized controlled trial (RCT) to obtain the group difference and standard deviation (SD) of the Quality of Recovery-15 (QoR-15) score, which is an indicator in perioperative clinical trials used to assess patient comfort and pain postoperatively [16,17], between M-TAPA and OSTAPB. We also evaluated the plasma concentrations of plain levobupivacaine in these two groups.

## 2. Materials and Methods

### 2.1. Study Design and Participants

This pilot randomized controlled trial was approved by the Institutional Review Board of the Nara Medical University Ethics Committee (Kashihara, Nara, Japan No. 2853; approved on 5 February 2021) and registered in the UMIN Clinical Trials Registry on 16 February 2021 (UMIN000043336, https://center6.umin.ac.jp/cgi-open-bin/icdr_e/ctr_view.cgi?recptno=R000049412) (accessed on 22 January 2024). Female patients aged 20–75 years with an American Society of Anesthesiologists physical status of 1–2 who were scheduled for TLH with four abdominal ports were enrolled in this study. The abdominal ports were placed at the following positions: one at the umbilicus and three below the umbilicus. Patients with an American Society of Anesthesiologists physical status classification ≥ 3, difficulty in communicating, coagulation disorders, skin disease at the puncture site, body weight ≤ 42 kg, body mass index ≥ 35 kg/m^2^, allergy to local anesthetics, anticoagulation therapy, or chronic opioid use were excluded. This trial was presented in accordance with the Consolidated Standards of Reporting Trials (CONSORT) guidelines. Written informed consent was obtained from all participants. The patients that were scheduled to undergo TLH were randomly assigned to the M-TAPA and OSTAPB groups by block randomization using GraphPad QuickCalcs (GraphPad, https://www.graphpad.com/quickcalcs/randomize1/) (accessed on 18 Feburary 2021). Patients and anesthesiologists were blinded to which nerve block was performed.

### 2.2. Ultrasound-Guided Block Procedures

After the induction of anesthesia, M-TAPA or OSTAPB was performed using the linear probe (6–13 MHz) of EDGE II (Fujifilm Sonosite, Tokyo, Japan) with the patients in a supine position. These procedures were performed by three anesthesiologists skilled in the nerve block (NT, TS, and YK).

#### 2.2.1. M-TAPA

In the M-TAPA group, a probe was placed on the notch between the ninth and tenth costal cartilages in the sagittal plane. The costal cartilage, external and internal oblique muscles, and transversus abdominis muscles were simultaneously delineated on the screen. A 20-gauge Tuohy needle (UNIEVER disposable nerve blockade needle; Huber; UNISIS, Tokyo, Japan) was inserted using an in-plane technique as described in our previous reports [11]. After confirming negative aspiration, 25 mL of 0.25% levobupivacaine was administered per side between the internal oblique muscle and the transversus abdominis muscle at the lower aspect of the 10th costal cartilage. The needle tip was positioned below the costal cartilage [18].

#### 2.2.2. OSTAPB

In the OSTAPB group, the probe was placed inferiorly, parallel to the costal margin, and scanned along the oblique subcostal line. The volume of the local anesthetics used was the same as that in the M-TAPA group. The local anesthetic was injected between the internal abdominis and transversus abdominis muscles, from the outside of the linear semilunaris to the anterior portion of the iliac crest. The method followed for performing OSTAPB was as described previously [19].

### 2.3. Intraoperative Procedure

After preoxygenation with 100% oxygen, anesthesia was induced with 2 mg/kg of propofol based on the ideal body weight calculation. Anesthesia was maintained using sevoflurane. Fentanyl and rocuronium were administered at the discretion of each anesthesiologist. Remifentanil (0.05–0.3 μg/kg/min) and sevoflurane flow rates were also determined at their discretion. An ultrasound-guided nerve block was performed with 25 mL of 0.25% levobupivacaine per side (total volume, 50 mL) after anesthesia induction. The plasma concentration of levobupivacaine (PC_levo_) in arterial blood samples obtained 15, 30, 45, 60, and 120 min after nerve block completion was measured. The anesthesiologist administered intravenous patient-controlled analgesia (IV-PCA) using a mechanical infusion pump (CADD-Solis 2110, Smiths Medical Japan Ltd., Tokyo, Japan) that was started with fentanyl 1500 μg and droperidol 5 mg mixed with 0.9% isotonic saline to a total volume of 100 mL from the end of the surgery, which was set at a background infusion rate of fentanyl 15 μg/h, a bolus dose of 15 μg on demand, and a lockout interval of 10 min.

### 2.4. Postoperative Management

The decision to discontinue IV-PCA was made by the gynecologist. Acetaminophen (15 mg/kg or not to exceed 1000 mg) was administered every 6 h immediately after returning to the ward until the second postoperative day (POD), and flurbiprofen infusion was administered if postoperative pain was not controlled (numerical rating scale, (NRS, 0–10) ≥4). Routine oral loxoprofen administration was initiated on the evening of POD 2.

### 2.5. Postoperative Plasma Levobupivacaine Measurement

The collected samples were immediately centrifuged at 1500 m/s^2^ at 4 °C for 10 min, and the plasma samples were stored at −20 °C until measurement. The plasma concentration was measured in the same manner as described in the previous report by Maruishi Pharmaceutical Corporation (Osaka, Japan) [15]. Maruishi Pharmaceutical Corporation, which produces and distributes levobupivacaine in Japan, provided non-financial support to our hospital by measuring the PC_levo_ in patient samples without compensation. The company was not involved in this study.

### 2.6. Outcomes

The primary outcome was the changes in the QoR-15 scores on POD 1 and 2 from the preoperative baseline, calculating the group differences and SDs between M-TAPA and OSTAPB. The QoR-15 questionnaire is recommended for assessing postoperative patient comfort and pain in perioperative clinical trials [15,17]. It consists of five domains related to quality of care: pain, physical comfort, physical independence, psychological support, and emotional state [16]. The questionnaire used an 11-point NRS, with scores ranging from 0 (extremely poor recovery) to 150 (excellent recovery).

The secondary outcomes were resting and dynamic pain scores (NRS, 0–10) at 2 h after surgery, POD 1, and POD 2; the PC_levo_ 15, 30, 45, 60, and 120 min after the initial intraoperative levobupivacaine bolus injection; comparison of the peak plasma concentration (C_max_) and time to peak plasma concentration (T_max_) for levobupivacaine; procedure duration (from skin preparation to nerve block completion); and postoperative fentanyl consumption. The C_max_ and T_max_ values of levobupivacaine were directly recorded for each patient based on the measured values.

Several literature reports and the FDA guidance (https://www.fda.gov/media/71512/download) (accessed on 22 January 2024) suggest that Bayesian analysis is useful for investigating the benefit of a new drug or medical device in pilot studies before conducting full-scale RCTs [20,21]. It is recommended that Bayesian posterior probability is used to make a Go/No-Go decision for future clinical studies rather than the “traditional” 5% significant difference with a limited sample size [20,21]. We also calculated the Bayesian posterior probability as post hoc analysis in this study.

### 2.7. Statistical Analysis

The primary outcomes (i.e., changes in QoR-15 scores on POD 1 and 2 from the preoperative baseline) were evaluated using a mixed model for repeated measurements and estimated mean with a 95% confidence interval (CI) and SD.

NRS scores for pain at each time point were compared using an unpaired *t*-test and estimated mean with 95% CI or chi-square test and estimated proportion with 95% CI, respectively. The PC_levo_ values at each time point and C_max_ values were compared using an unpaired *t*-test, and the estimated mean with 95% CI and T_max_ were compared using the chi-square test. Intraoperative data were evaluated using an unpaired *t*-test. The post hoc analysis included calculation of sample size for full-RCT based on primary endpoints, examination of QoR-15 for each item and distribution of patients by QoR-15 score category using Fisher’s exact test [22], and calculation of Bayesian posterior probability for full-RCT. All statistical analyses were performed using SAS version 9.4 (SAS Institute Japan Ltd., Tokyo, Japan).

### 2.8. Sample Size Calculation

M-TAPA is a novel block; hence, no study has currently compared it with OSTAPB using QoR-15 in TLH. The main objective of this pilot study was to obtain between-group differences and SDs of QoR-15 scores as reference values for efficacy assessment. The effect size was assumed to be 0.63 with reference to a previous study [23]. The recommended sample size for a pilot trial of a full RCT designed with 90% power and a two-sided 5% significance threshold is 15 per group [24]. A dropout rate was set at 20%, with 20 participants in each group.

## 3. Results

### 3.1. Recruitment and Exclusions

**Figure 1:**
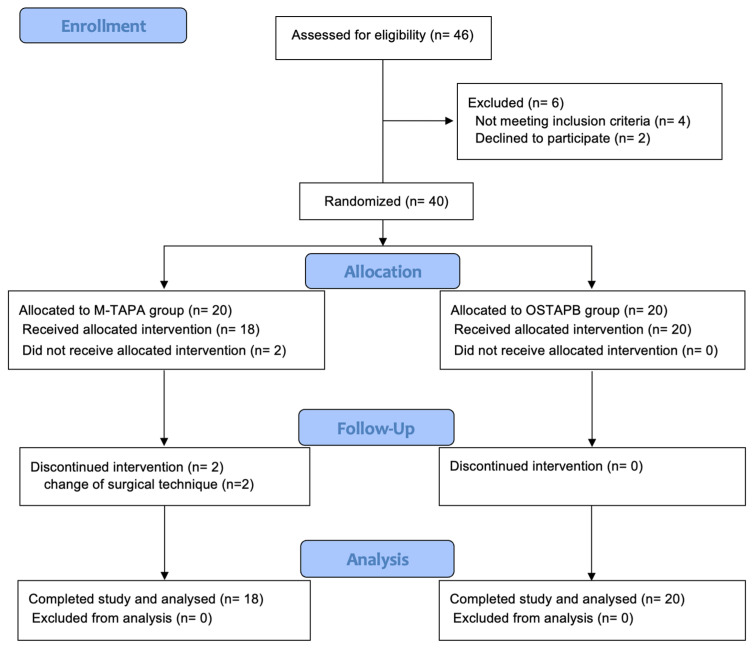
Patient flow diagram. M-TAPA, modified thoracoabdominal nerves block through the perichondrial approach; OSTAPB, oblique subcostal transversus abdominis plane block.

The CONSORT flowchart for this study is presented in Figure 1. Among the 46 eligible patients screened between March 2021 and June 2022, 6 were excluded before randomization (4 patients did not meet the inclusion criteria, and 2 declined participation). Subsequently, 40 patients were randomized into two groups. After randomization, two patients were excluded due to changes in the surgical technique (from laparoscopic to open surgery). In total, 38 patients (18 in the M-TAPA group and 20 in the OSTAPB group) were included in the study.

### 3.2. Patient Characteristics and Intraoperative Data

Patient characteristics, including intraoperative data, are presented in Table 1. There were no complications such as hematoma or local anesthetic systemic toxicity.

### 3.3. Primary and Secondary Outcomes

**Figure 2:**
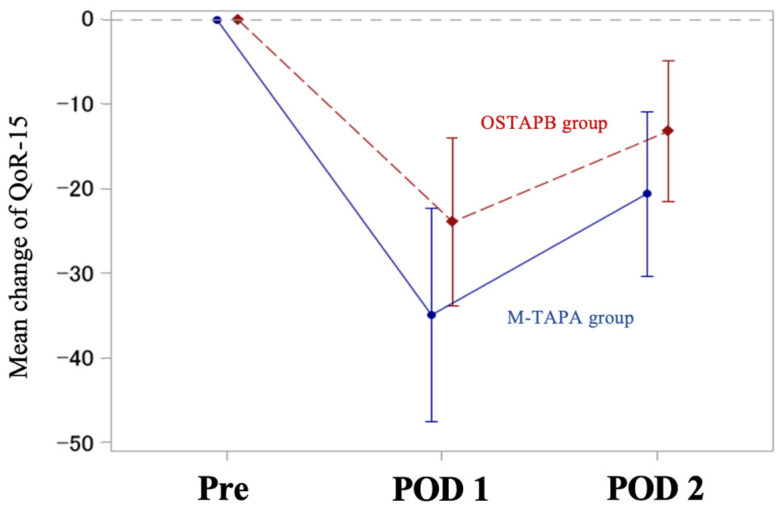
Mean changes in the QoR-15 scores from the preoperative baseline on postoperative day 1 and 2. The error bars represent 95% CI. Values in the table are expressed as mean with 95% CI. Difference between groups from baseline = M-TAPA minus OSTAPB are estimated using mixed model for repeated measurement. CI, confidence interval; M-TAPA, modified thoracoabdominal nerves block through perichondrial approach; OSTAPB, oblique subcostal transversus abdominis plane block; POD, postoperative day; Pre, preoperative day; QoR-15, Quality of Recovery-15.

**Figure 3:**
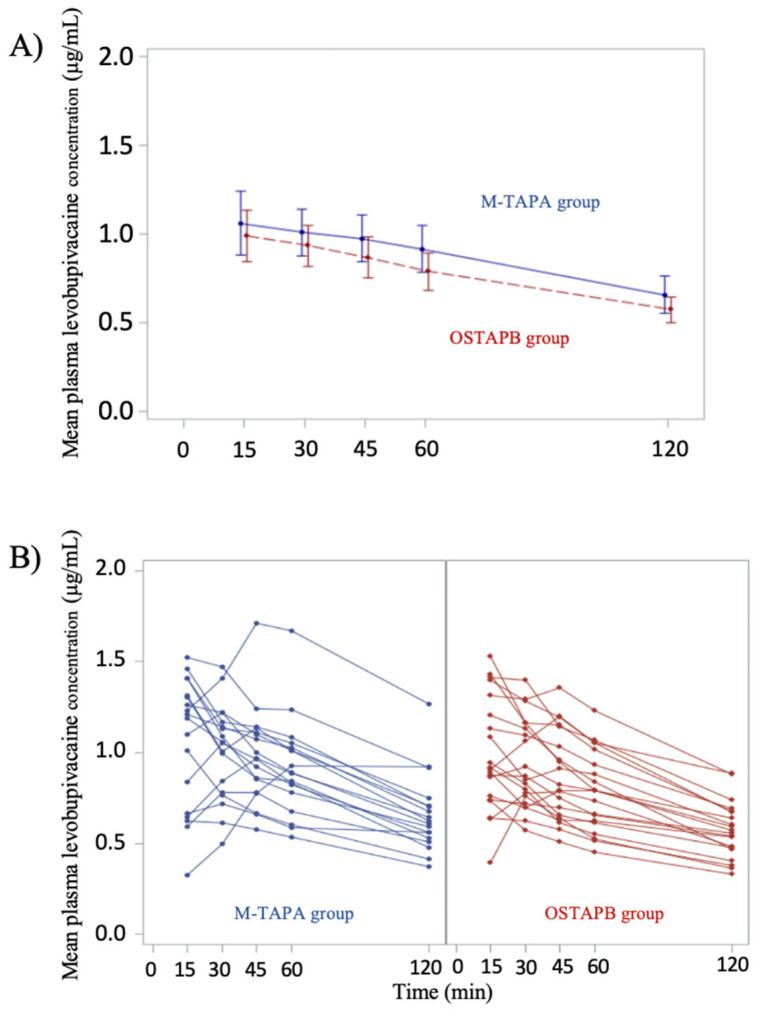
Comparison of the plasma levobupivacaine concentrations between both groups. (**A**) Mean plasma levobupivacaine concentration 15, 30, 45, 60, and 120 min after performing nerve block. The error bars represent 95% CI. (**B**) Spaghetti plot for plasma levobupivacaine concentrations 15, 30, 45, 60, and 120 min after performing nerve block. CI, confidence interval; M-TAPA, modified thoracoabdominal nerves block through perichondrial approach; OSTAPB, oblique subcostal transversus abdominis plane block.

The mean changes in the QoR-15 scores between the two groups (M-TAPA—OSTAPB) on POD 1 and 2 from the preoperative baseline were −11.3 (95% CI; −24.9 to 2.4; SD, 22.8) and −7.0 (95% CI; −20.5 to 6.6; SD, 18.7), respectively (Table 2 and Figure 2). The mean C_max_ (µg/mL) of the M-TAPA and OSTAPB groups were 1.17 (95% CI; 1.03 to 1.32) and 1.04 (95% CI; 0.91 to 1.17), respectively. The T_max_ showed a peak at 15 min in the majority cases, with some cases showing a peak at 60 min; however, no cases in either group showed a peak at 120 min. There were no cases exceeding the toxic level (2.62 μg/mL) [25] (Figure 3). The mean NRS for pain in the M-TAPA and OSTAPB groups at 24 h postoperatively was 1.6 and 1.3 at rest (*p* = 0.49); 3.0 and 3.4 at movement (*p* = 0.54), respectively. The time in which to perform the nerve block was shorter in the M-TAPA group than in the OSTAPB group (4.7 vs. 8.9, *p* < 0.001).

### 3.4. Post Hoc Analysis

Setting a power of 90%, with α = 0.05 (two-sided) as described in the sample size calculation, and referring the group difference and SD in the changes from baseline in the QoR-15 scores on POD 1, we calculated the sample size required for future clinical studies to be 87 subjects per group. In POD 2, the same calculation showed that 151 subjects per group would be needed. We also calculated the Bayesian posterior probability regarding the probability that the full-RCT would yield our initial hypothesis of “less reduction in QoR-15 for M-TAPA than for OSTAPB.” When the minimal clinically important difference (MCID) in QoR-15 changes was set to 8.0 [26], the Bayesian posterior probability (i.e., the probability that the group difference M-TAPA—OSTAPB in the full-RCT would be +8.0 or greater) was 19.9% for POD 1 and 21.1% for POD 2 (Figure S1). Since the MCID of QoR-15 scores was set to 6.0 in the other report [27], we also calculated the Bayesian posterior probability, and found it to be 22.4% for POD 1 and 24.4% for POD 2. (Figure S2) In contrast, the Bayesian posterior probability that the decrease in QoR-15 is less for M-TAPA (i.e., the probability that the difference between groups is greater than 0 in full-RCT) was 31.0% for POD 1 and 35.4% for POD 2. A QoR-15 item-by-item examination revealed that OSTAPB was significantly less painful than M-TAPA in the “moderate pain” section (M-TAPA: 5.2 ± 3.2, OSTAPB: 7.4 ± 1.7; *p* = 0.012) (Table 3). There were no significant differences in the distribution of patients by QoR-15 category (Table S1). 

## 4. Discussion

This study is the first pilot RCT on the efficacy of M-TAPA in TLH. Although a retrospective study has examined the efficacy of M-TAPA in gynecologic laparoscopic surgery, this was not a targeted study specific to TLH because it included four different types of surgical procedures [28]. Previously, Tran et al. suggested that the newer truncal block should be compared with the TAP block [5]. However, the RCTs on M-TAPA included laparoscopic cholecystectomy and laparoscopic inguinal hernia repair, and in these reports suggesting that M-TAPA was effective, the majority of the patients included in the control group were with wound infiltration or no intervention [12,13,14]. Thus, in this study, we compared the quality of recovery and local anesthetic plasma concentration level of M-TAPA and OSTAPB.

In this pilot study, we obtained the group differences (M-TAPA-OSTAPB) and SDs in the changes from baseline in the QoR-15 scores on POD 1 and 2. The plasma levobupivacaine concentration levels remained below the toxic threshold of 2.62 μg/mL in the M-TAPA group and followed the same trend as that in OSTAPB group.

The sample size for future clinical studies calculated from these results is estimated to be at least 65 subjects per group, which would require a lot of resources to conduct a full-RCT. Even if a full-RCT were performed, the Bayesian posterior probability that the initial hypothesis “M-TAPA reduce the change of QoR-15 scores less than OSTAPB in TLH with clinical significance” would be reached in a full-RCT ranged from 19.9 to 24.4%. The Bayesian posterior probability that M-TAPA had an equal or slightly lower change in QoR-15 than OSTAPB was 31.0% for POD 1 and 35.4% for POD 2. According to a previous study, the former probability (group difference is more than MCID; efficacy criterion) should be greater than 50%, and the latter probability (group difference is more than 0; precision criterion) should be greater than 95% [29]. Lee proposed a Go/No-Go hurdle for future clinical study such as “whether a mean difference greater than the MCID can be obtained with at least 75% certainty,” but even this criterion was not met. Therefore, there may be little need to conduct a full-RCT under the hypothesis that “M-TAPA has less QoR-15 change than that in OSTAPB in TLH.” For the new peripheral nerve blocks, the same as for the drugs and medical devices, efficacy should be confirmed in pilot studies before proceeding to clinical trials, and Bayesian statistics could be used to make future Go/No-Go decisions [21].

There is a further finding suggested by the pilot study. Post hoc analysis of each QoR-15 subscale item showed that there was a large difference in the moderate pain between both groups. OSTAPB involves injecting local anesthetics continuously into the layers of the transversus abdominis plane, providing a certain analgesic range from the T7 to T12 anterior cutaneous branches. This study was designed and conducted based on initial reports that M-TAPA provided overnight analgesia with a maximum analgesic range of T3-T12 [9]. M-TAPA has recently been reported to be effective for the T8–T11 branches and less than 60% effective in the T12 [11,30,31,32], suggesting that there might be cases in which the caudal port wound was not covered. Furthermore, M-TAPA was reported to provide long duration of analgesia; however, a recent study has indicated that the median duration of analgesia after M-TAPA was 10–20 h [25], although after OSTAPB, it was reported to be 6–18 h [7]. These findings in recent studies may support our results, which could not show superiority of M-TAPA over OSTAPB. We also expected T_max_ to be slower in the M-TAPA group than in the OSTAPB group due to overnight analgesia, which was initially reported [11,13]. However, changes in PC_levo_ showed a similar pattern in both groups, and the C_max_ and T_max_ of PC_levo_ showed no large differences between the two groups. The study by Aikawa et al. using the same dose of levobupivacaine as that used in our study was characterized by the addition of epinephrine, and these two findings revealed the effect of epinephrine in delaying T_max_ and lowering C_max_ [15]. In this study, M-TAPA showed a significant advantage regarding the duration to perform the procedure. The plasma local anesthetic concentration kinetics of M-TAPA were also clarified, suggesting that these results provide multiple options (e.g., preoperative or postoperative) for when to perform M-TAPA in perioperative management. M-TAPA may be the alternative not only in cases where OSTAPB is hesitated (e.g., skin lesions at the puncture site) but also in operating room management.

Our study has some limitations. First, the area of sensory loss was not evaluated after M-TAPA or OSTAPB. Second, the decision to discontinue IV-PCA fentanyl was made by the treating gynecologists. This fact might have affected the amount of fentanyl usage postoperatively. Third, intraoperative opioid administration was not strictly standardized and was left to the discretion of the anesthesiologist. Finally, the time points at which blood samples were collected were not sufficient to accurately determine the C_max_ and T_max_ because of medical safety considerations for the prevention of iatrogenic anemia.

In conclusion, M-TAPA may not be superior to OSTAPB for TLH in terms of QoR.

## Figures and Tables

**Table 1:** Patient characteristics and intraoperative data.

Patient Characteristics	M-TAPA (n = 18)	OSTAPB (n = 20)
Age (years)	46.5 (±8.5)	51.6 (±10.4)
Body mass index (BMI)	22.8 (±2.9)	23.9 (±4.3)
ASA-PS 1/2	9/9	4/16
Albumin (g/dL)	4.4 (±0.4)	4.5 (±0.3)
AST (IU/L)	22.9 (±9.3)	22.2 (±9.8)
ALT (IU/L)	21.5(±16.4)	23.15(±16.6)
Total Bilirubin (mg/dL)	0.7 (±0.3)	0.7 (±0.3)
PT-INR	1.0 (±0.1)	0.9 (±0.1)
APTT (second)	26.8 (±3.3)	29.9 (±3.2)
Platelet (×10^4^/µL)	23.5 (±4.6)	26.8 (±7.6)
Intraoperative data	M-TAPA (n = 18)	OSTAPB (n = 20)
Duration of anesthesia (min)	203.6 (±24.3)	211.4 (±37.7)
Duration of surgery (min)	142.8 (±22.3)	147.0 (±34.2)

Data are presented as mean (±SD). ASA-PS, American Society of Anesthesiologists physical status; AST, aspartate aminotransferase; ALT, alanine aminotransferase; PT-INR, prothrombin time-international normalized ratio; APTT, activated partial thromboplastin time; M-TAPA, modified thoracoabdominal nerves block through perichondrial approach; OSTAPB, oblique subcostal transversus abdominis plane block; SD, standard deviation.

**Table 2:** Primary and secondary outcomes.

Primary Outcome					
	The changes of Quality of Recovery-15 from baseline (M-TAPA—OSTAPB)				
		POD 1—Pre	−11.3 (95% CI, −24.9 to 2.4; SD, 22.8)		
		POD 2—Pre	−7.0 (95% CI, −20.5 to 6.6; SD, 18.7)		
**Secondary Outcomes**			**M-TAPA (n = 18)**	**OSTAPB (n = 20)**	***p*-Value**
Plasma levobupivacaine concentration (µg/mL)					
		15 min	1.06 [0.88 to 1.24]	0.99 [0.84 to 1.14]	0.510
		30 min	1.01 [0.88 to 1.14]	0.93 [0.82 to 1.05]	0.365
		45 min	0.97 [0.84 to 1.11]	0.87 [0.75 to 0.98]	0.203
		60 min	0.92 [0.78 to 1.05]	0.79 [0.69 to 0.89]	0.115
		120 min	0.66 [0.55 to 0.76]	0.57 [0.50 to 0.65]	0.172
	Cmax (µg/mL)		1.17 [1.03 to 1.32]	1.04 [0.91 to 1.17]	0.153
	Tmax, n (%)				0.690
		15 min	11 (61)	14 (70)	
		30 min	4 (22)	2 (10)	
		45 min	2 (11)	3 (15)	
		60 min	1 (6)	1 (5)	
		120 min	0 (0)	0 (0)	
NRS at rest					
		2 h	2 [1–3]	2 [1–3]	0.492
		24 h	1 [1–3]	1 [0–2.5]	0.491
		48 h	1 [0–3]	1 [0–2.5]	0.693
	at movement				
		2 h	3 [2–5]	3 [2–4]	0.411
		24 h	3 [2–4]	3 [2–4.5]	0.540
		48 h	3 [1–4]	3 [3–6]	0.228
Intraoperative fentanyl use (µg/kg)			3.8 [3.1 to 4.5]	3.8 [3.2 to 4.5]	0.944
Intraoperative remifentanil use (mg/kg)			0.030 [0.024 to 0.035]	0.035 [0.029 to 0.041]	0.183
Postoperative fentanyl use within 48 h (µg/kg)			7.8 [6.4 to 9.2]	7.6 [6.5 to 8.6]	0.764
Time to perform nerve block (min)			4.7 [3.9 to 5.5]	8.9 [8.0 to 9.8]	<0.001

Values are expressed as the mean with a 95% confidence interval or the count with proportion. Scores of NRS are expressed as the median (IQR). POD 1 and 2 refer to 24 and 48 h after surgery, respectively. C_max_, the peak plasma concentration of levobupivacaine; M-TAPA, modified thoracoabdominal nerve block through perichondrial approach; NRS, numerical rating scale; OSTAPB, oblique subcostal transversus abdominis plane block; POD, postoperative day; Pre, preoperative day; T_max_, the time to peak plasma concentration of levobupivacaine.

**Table 3:** Comparison of global and itemized Quality of Recovery-15.

QoR-15 Global Score		M-TAPA	OSTAPB	*p*-Value
		Pre, POD 2; n = 18 POD 1; n = 17	n = 20	
	Pre	145.9 (±6.3)	143.4 (±6.9)	0.247
	POD 1	110.9 (±24.6)	119.5 (±21.8)	0.269
	POD 2	125.3 (±20.9)	130.3 (±18.7)	0.449
**QoR-15 Subscale**				
POD 1		n = 17	n = 20	
	1. Able to breathe easily	9.3 (±1.4)	9.5 (±1.2)	0.544
	2. Been able to enjoy food	5.7 (±3.6)	6.3 (±3.6)	0.615
	3. Feeling rested	6.4 (±3.1)	7.7 (±3.1)	0.232
	4. Have had a good sleep	7.7 (±2.4)	7.5 (±3.1)	0.785
	5. Able to look after personal toilet and hygiene unaided	7.7 (±2.8)	8.1 (±3.2)	0.696
	6. Able to communicate with family or friends	7.8 (±2.5)	8.9 (±2.1)	0.147
	7. Getting support from hospital doctors and nurses	9.7 (±0.9)	9.9 (±0.5)	0.375
	8. Able to return to work or usual home activities	5.9 (±3.7)	6.9 (±4.1)	0.486
	9. Feeling comfortable and in control	5.7 (±3.2)	5.9 (±3.5)	0.821
	10. Having a feeling of general well-being	6.9 (±2.5)	7.0 (±3.2)	0.944
	11. Moderate pain	5.2 (±3.2)	7.4 (±1.7)	0.012
	12. Severe pain	9.2 (±1.4)	9.1 (±1.8)	0.803
	13. Nausea or vomiting	7.2 (±3.8)	8.4 (±2.4)	0.264
	14. Feeling worried or anxious	7.7 (±2.5)	7.7 (±2.4)	0.994
	15. Feeling sad or depressed	9.1 (±1.6)	9.6 (±0.8)	0.238
POD 2		n = 18	n = 20	
	1. Able to breathe easily	9.5 (±1.3)	9.7 (±0.8)	0.652
	2. Been able to enjoy food	7.8 (±2.7)	8.7 (±2.5)	0.336
	3. Feeling rested	8.6 (±1.9)	8.5 (±2.1)	0.865
	4. Have had a good sleep	6.7 (±2.8)	6.9 (±3.1)	0.850
	5. Able to look after personal toilet and hygiene unaided	9.9 (±0.2)	9.7 (±1.2)	0.307
	6. Able to communicate with family or friends	8.7 (±2.7)	9.9 (±0.5)	0.064
	7. Getting support from hospital doctors and nurses	9.9 (±0.2)	9.8 (±0.6)	0.356
	8. Able to return to work or usual home activities	7.4 (±3.1)	9.0 (±2.2)	0.089
	9. Feeling comfortable and in control	8.0 (±2.0)	8.0 (±2.6)	1.000
	10. Having a feeling of general well-being	8.1 (±2.0)	8.2 (±2.8)	0.961
	11. Moderate pain	6.2 (±3.1)	6.3 (±2.6)	0.886
	12. Severe pain	8.9 (±1.5)	8.8 (±2.2)	0.820
	13. Nausea or vomiting	8.6 (±2.4)	8.8 (±2.6)	0.812
	14. Feeling worried or anxious	8.0 (±2.5)	9.2 (±1.9)	0.111
	15. Feeling sad or depressed	9.0 (±1.5)	9.3 (±1.4)	0.589

Values are expressed as mean with standard deviation in QoR-15 global scores and each item of QoR-15. POD 1 and 2 refer to 24 and 48 h after surgery, respectively. M-TAPA, modified thoracoabdominal nerve block through perichondrial approach; OSTAPB, oblique subcostal transversus abdominis plane block; POD, postoperative day; Pre, preoperative day; QoR-15, Quality of Recovery-15.

## Data Availability

The data associated with the paper are not publicly available but are available from the corresponding author upon reasonable request.

## Supplementary Materials

The following supporting information can be downloaded at https://doi.org/10.5281/zenodo.10397827 (accessed on 22 January 2024). Figure S1: Bayesian posterior probability of full-randomized controlled trial based on mean and standard deviation of postoperative day 1 for the hypothesis ‘M-TAPA has a smaller decrease in QoR-15 than OSTAPB’. Figure S2: Bayesian posterior probability of full-randomized controlled trial based on mean and standard deviation of postoperative day 2 for the hypothesis ‘M-TAPA has a smaller decrease in QoR-15 than OSTAPB’. Table S1: Comparison of distribution of patients according to categories of Quality of Recovery-15 score.
